# Utilization of adolescent health services during the COVID-19 pandemic: evidence on impact and adaptations from a rapid assessment survey in the Philippines

**DOI:** 10.1186/s12889-023-15102-2

**Published:** 2023-03-14

**Authors:** Adeyemi Okunogbe, Meagan Meekins, Khalida Saalim, Mary Angeli Conti-Lopez, Rosario Marilyn Benabaye, Ophelia M. Mendoza, Rio Julio, Laurentiu Stan, Cristina Bisson

**Affiliations:** 1 Global Health Division, RTI International, 701 13th Street, N.W., Suite 750, 20005 Washington, DC USA; 2 Global Health Division, RTI International, Research Triangle Park, NC USA; 3 USAID ReachHealth Project, RTI International, Metro Manila, Philippines

**Keywords:** Adolescent sexual and reproductive health, Adolescent mental health, COVID-19 pandemic, Health utilization, Philippines, Low and middle income countries, Telemedicine

## Abstract

**Background:**

Due to the COVID-19 pandemic, many challenges in adolescent health have been exacerbated including increased cases of early marriages, domestic violence, higher rates of anxiety and depression, and reduced access to sexual and reproductive health services for adolescents. This study examines the impacts of the pandemic on adolescent health services utilization and potential adaptations in the Philippines.

**Methods:**

The data used in this study was from a rapid telephone assessment survey of 148 adolescent-friendly health facilities (rural health units) in the Philippines. We employed a mixed-methods research approach comprising both quantitative and qualitative analyses in three phases. First, we conducted a descriptive analysis of the status of adolescent healthcare access and utilization during COVID-19. Next, we examined using multivariate ordered logistic regressions how staff availability and adolescent health (AH) service provision modalities influenced AH service utilization in terms of the average number of adolescents served per week during compared to before the pandemic. We also conducted a complementing qualitative analysis of the challenges and corresponding adaptive solutions to ensuring continuity of AH services in facilities.

**Results:**

We find that two months into the pandemic, 79% of adolescent-friendly trained staff were reporting for duty and 64% of facilities reported no staff disruptions. However, only 13% of facilities were serving the same number of adolescents or greater than before COVID-19. The use of more modalities for AH service provision (including telehealth) by facilities was significantly associated with increased likelihood to report serving the same number of adolescent or greater than before COVID-19 compared to those who used only one modality.

**Conclusion:**

Investments in multiple modalities of care provision, such as telehealth could improve AH services utilization and help sustain connection with adolescents during shocks, including future outbreaks or other stressors that limit physical access to health facilities.

## Background

There is increasing recognition globally of the need to invest in adolescent health based on growing evidence of the multi-generational outcomes of poor access and quality of care in this vulnerable population [1, 2]. Over the last three decades, there has been significant decline in adolescent pregnancy, child marriage and female genital mutilation as well as increased funding for programs and research targeting adolescents [3]. While great progress has been made, several critical gaps remain—including increasing reports of intimate partner violence, substantial inequalities in access and quality within and across countries, and resistance due to unhealthy social and cultural norms around adolescent health (AH) [3–5]. This study examines the impact of the COVID-19 pandemic on adolescent health services (AHS) utilization as well as potential adaptive strategies across a sample of adolescent-friendly health facilities in the Philippines.

### Context

The Philippines is an archipelagic nation in Southeast Asia of over 7,500 islands across three island groups: Luzon, Visayas, and Mindanao. The Philippines is a lower-middle income country and has a total population of 111 million, with about 19% of the population between 15 and 24 years old [6, 7]. The country has experienced substantial improvement in health status over the last few decades [8, 9]. The nation has a total fertility rate of 2.78, adolescent fertility rate of 56 per 1000 women, maternal mortality ratio of 121 deaths per 100,000 live births, and a life expectancy at birth of 70 years [9–12]. However, there are substantial variations across regional, urban–rural and socioeconomic status [8]. The health system is decentralized with barangay (village) health stations and local health centers at the lowest level of the health system for primary care delivery. The country has recorded modest progress in key AH indicators such as increase in use of health facilities, including antenatal care visits; delivering children in facilities; accessing postnatal care; and receiving contraceptive methods in the public facilities among ages 15–24 [13, 14]. Adolescent pregnancies and births among youth is slowly declining, while evidence on knowledge and exposure to sexual and reproductive health information are mixed [13, 14]. .

### COVID-19 pandemic in the Philippines

The Philippines confirmed its first two cases of COVID-19 on January 27, 2020 and as of January 2023 had reported approximately 4 million confirmed cases and over 65,000 deaths [15]. The Philippine Government enacted a community quarantine order on March 15th, 2020 that required children, adolescents and older adults to stay in their homes [16].

The COVID-19 pandemic has brought an unparalleled set of challenges to health systems with far reaching health, social and economic impacts around the globe. Early pandemic responses including lockdowns, travel restrictions and other social distancing guidelines led to reduced access for non-COVID-19 related conditions deemed non-essential due to factors such as patients fearing exposure to COVID-19 at healthcare facilities, disruptions in staffing, re-assignment of staff to COVID-19 wards, and lack of public transportation to appointments [17–19]. While a growing body of evidence has documented reduction in access and utilization of non-COVID-19 related health services especially in the early phase of the pandemic [20, 21], less have been documented about effects on AHS utilization [22, 23].

Potential impacts of the pandemic on AH include reductions in contraceptive use, STI screening/ treatment and sex education through channels such as limited privacy and confidentiality, reduced affordability and geographic accessibility [24–28]. Other research has also indicated negative impact of the pandemic on mental health of adolescents from school closures, quarantines and isolation including anxiety and depression as well abuse and gender violence [22, 23, 29–32].

### Research questions

We add to this growing body of evidence by investigating the impact of COVID-19 related restrictions on AHS access and utilization in the Philippines. Our research questions are (1) What was the status of AHS access and utilization during COVID-19 response compared to before? (2) What were the most common psychosocial concerns identified among adolescents before and during COVID response? (3) How did staffing availability and service provision modalities influence adolescent health service utilization during the pandemic? (4) What were the challenges and corresponding adaptive solutions to ensuring continuity of FP services in facilities?

Answering these questions is relevant for deepening understanding of COVID-19 impacts and regaining pre-COVID-19 pandemic momentum for adolescent health in the Philippines as well as in strengthening the health system for future shocks, including future infectious disease outbreaks. This will contribute to the policy dialogue on how health systems in low- and middle-income countries can adapt to disruptions from shocks and unanticipated stresses that can have devastating impact on adolescents and communities.

## Methods

### Setting and sample

The data used in this study was from a rapid assessment survey of all adolescent-friendly rural health units across 32 project sites (provinces/chartered cities) in 11 regions covering the three island groups of Philippines (Mindanao, Luzon and Visayas). These adolescent-friendly facilities have providers who have been adequately trained, services that are accessible and an environment within the facility that provides audio and visual privacy for adolescents [33]. The facilities were surveyed on the 4th and 5th of May 2020 (two months into the pandemic) as part of an assessment to inform the USAID ReachHealth program (a five-year project working to reduce unmet needs for family planning (FP) services and decrease teen pregnancies and newborn morbidity and mortality), and so only focused on the sites served by the program. There were two sets of selection criteria for included facilities, the first is that the relevant Provincial or City Health Officer (P/CHO) had to give permission for health facilities within their areas to be contacted for the survey, given the high burden of COVID-19 response on the health system. If they could not be reached, or did not give permission, AFHFs in that area/site were excluded. The second is that the team asked if the health facility was still up to date on AFHF certification requirements including (1) having at least one adolescent friendly health trained staff, (2) having a place for counseling or offering services for adolescents with audio-visual privacy and (3) a referral tool for adolescent services. If a facility no longer fulfilled any of these three elements of adolescent friendly care, it was excluded from the sample. The final sample included facilities from all sites except Cavite, Iloilo, Iloilo City and Mandaue City, whose P/CHOs could not be reached. There were 199 RHU facilities that provided adolescent reproductive health (ARH) services in the study sites out of which 179 agreed to participate and 148 met the two criteria. Interviews were conducted by telephone and in English by technical associates on the USAID ReachHealth program. The survey questions were close-ended except for the last two questions on challenges and adaptations that were open-ended. The average duration of the interviews was about an hour and responses were collated in Microsoft Forms and stored in a secured organization SharePoint site. We employed a mixed-methods research approach comprising both quantitative and qualitative analyses in three phases.

### Quantitative analysis

In the first phase, we conducted a descriptive analysis of the status of AHS access and utilization before and during COVID-19 response for the 148 RHUs in the study. We explored AHS access and utilization using the following variables: operating capacity in terms of number of hours per day, average number of adolescents served per week, number of staff trained in adolescent-friendly care reporting for duty during pandemic compared to before pandemic. The response categories for these three variables were: Much less than before, less than before, same as before, and greater than before. Another variable (binary) analyzed was whether the facility experienced disruption in usual staff that provided AHS (Yes/No). In addition, we conducted a descriptive analysis of the most common psychosocial concerns identified among adolescents who sought care before and during the pandemic. Health facility representatives were asked to select among a group of psychosocial issues namely: Early Sexual Engagement, Anxiety and Depression, Dysfunctional Family, Gender Based Violence, Sexual Abuse, Smoking/Tobacco/Vaping Use, Bullying, Suicidal Ideation, Drug Use and Others. These issues were then ranked by number of mentions (how many facilities flagged such a concern as common) for each time period (before and during pandemic) and compared. This descriptive analysis was restricted to 84 RHUs that provided mental health services/psychosocial evaluation for adolescents both during and before the pandemic.

The second phase of the study is a retrospective cross-sectional analysis that examined how staff availability, staffing disruptions and service provision modalities influenced AHS utilization. The main outcome or dependent variable is AHS utilization measured as the average number of adolescents served per week during compared to before the pandemic. The independent variables are staff availability, staffing disruptions and service provision modalities. Service provision modalities refer to whether a health facility used any of these four modalities namely: clients come to the facility; service providers reach out to clients; community volunteers reach out to clients and telemedicine. From the binary responses (Yes/No) to the use of these modalities, we derived a secondary variable of the number of modalities a health facility used out of the four modalities. We also controlled for variation based on the island group where facility is located by including island -fixed effects.

*Statistical Analysis*: We employed multivariate regression to examine the associations between average number of adolescents served per week (as the dependent variable) and three explanatory variables-staff availability, staffing disruption and service provision modalities. Because the dependent variable is an ordinal variable (with responses: much less than before, less than before, same as before, and greater than before), we used an ordered logistic regression modelling approach. Our regression framework takes the form shown below:

$${\varvec{Y}}_{\varvec{i}}=\propto .{\varvec{S}}_{\varvec{i}} + {\varvec{\beta }.\varvec{D}}_{\varvec{i}} + {{\varvec{\delta }.\varvec{M}}_{\varvec{i}}+{\varvec{\phi }}_{\varvec{r}}+\varvec{\epsilon }}_{\varvec{i}} \left(1\right)$$

where ${Y}_{i}$denotes the ordinal dependent (outcome) variable (average number of adolescents served per week) for health facility *i*; ${S}_{i}$ is a categorical variable denoting whether the number of adolescent-friendly care trained staff reporting for duty is much less than before, less than before, same as before, or greater than before;${D}_{i}$ is an indicator/binary variable denoting whether or not the facility was experiencing staffing disruption during the pandemic; ${M}_{i}$ is a continuous variable denoting the number of modalities the facility was currently using to provide adolescent health services, ${\varvec{\phi }}_{\varvec{r}}$ captures island-group fixed effects and ${\varvec{\epsilon }}_{\varvec{i}}$ is the independently distributed error term. Standard errors are robust, and the analysis was conducted using *Stata/MP 15.1.* We hypothesized that a greater or same number of trained staff reporting for duty, no staffing disruption and utilizing more modalities of services would be associated with higher likelihood of serving more adolescents per week during the pandemic compared to before the pandemic.

### Qualitative analysis

The results from the 1st and 2nd phase were then complemented by the 3rd phase which was a qualitative analysis of the challenges and corresponding adaptive solutions to ensuring continuity of AHS in facilities. This was a thematic analysis conducted using Atlas.ti on the two open-ended questions asked during the survey. The first question is “what challenges is your facility facing during the pandemic?”, and the second is “what solutions have been considered or implemented for the challenges?” Interviewers took notes of responses provided by the respondents. A selection of the responses was read, and memos were created on emergent themes to create a codebook. Researchers MM and KS coded 25 out of the 148 set of responses together, resolved differences in code use, updated the codebook and split the rest of the responses. New themes were documented, and the codebook was updated as required. We sought to understand the most common challenges and solutions being discussed and how they aligned with our findings from the quantitative analyses.

This study was part of a quality assurance evaluation to assess the status of the DOH’s adolescent reproductive health programming under COVID-19 and thus did not require Ethical Review Board (ERB) approval.

## Results

### Phase I: descriptive results

**Fig. 1:**
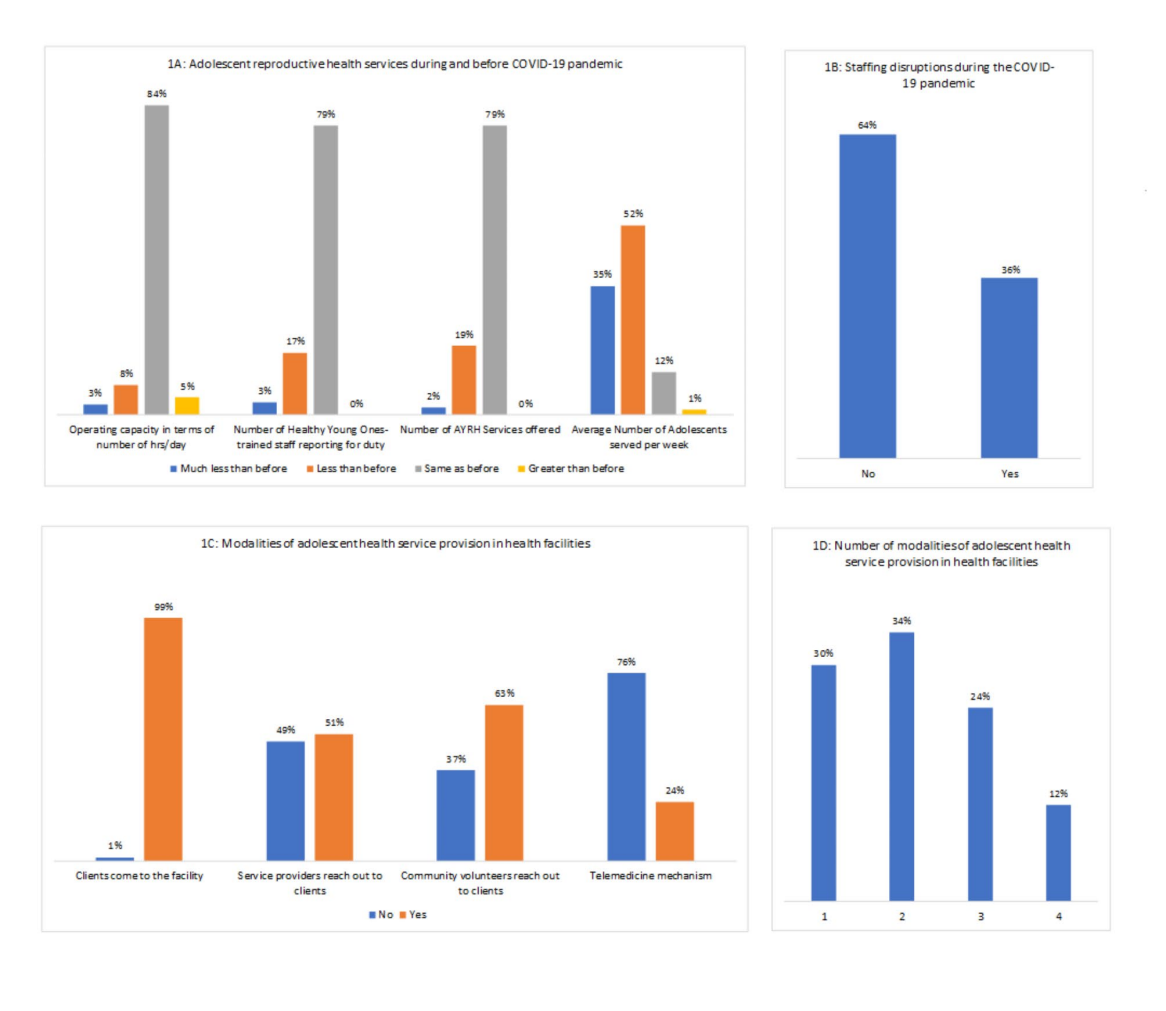
Descriptive summary of study variables (n = 148)

Figure 1 A-D shows the frequency distribution of study variables. 89% of health facilities were operating at the same number of hours or greater than they were before the pandemic started. 79% of facilities reported that the number of adolescent-friendly care trained staff reporting for duty were at the same level as before the pandemic while the remaining reported less or much less than before (Fig. 1 A). 36% of facilities noted that they were experiencing general staffing disruptions (Fig. 1B).

Only 13% of facilities reported serving the same or greater number of adolescents than they were before the pandemic, while the rest noted lower or much lower service utilization rate (Fig. 1 A). The modality most used by all facilities was for clients to come to the facility while the least used modality was telemedicine (Fig. 1 C). 70% of health facilities were using multiple modalities to serve adolescent clients during the pandemic (Fig. 1D), but we do not have data on how many modalities were used before the pandemic.

**Table 1:** Psychosocial concerns among adolescents before and during COVID-19 (n = 77)

Psychosocial Concern, identified by HEADSS assessment*	Before COVID − 19 Ranking *(Frequency of mentions)*	During COVID − 19 Ranking *(Frequency of mentions)*
Early Sexual Engagement	**1** *(58)*	**1** *(40)*
Anxiety and Depression	**4** *(34)*	**2** *(20)*
Dysfunctional Family	**5** *(33)*	**3** *(19)*
Sexual Abuse	**7** *(28)*	**4** *(16)*
Gender Based Violence	**7** *(28)*	**4** *(16)*
Smoking/Tobacco/Vaping Use	**2** *(45)*	**6** *(15)*
Bullying	**3** *(38)*	**7** *(14)*
Suicidal Ideation	**9** *(24)*	**7** *(14)*
Drug Use	**5** *(33)*	**9** *(13)*

*Home & Environment; Education & Employment; activities; drugs; sexuality; Suicide/Depression (HEADSS) assessment

**Fig. 2:**
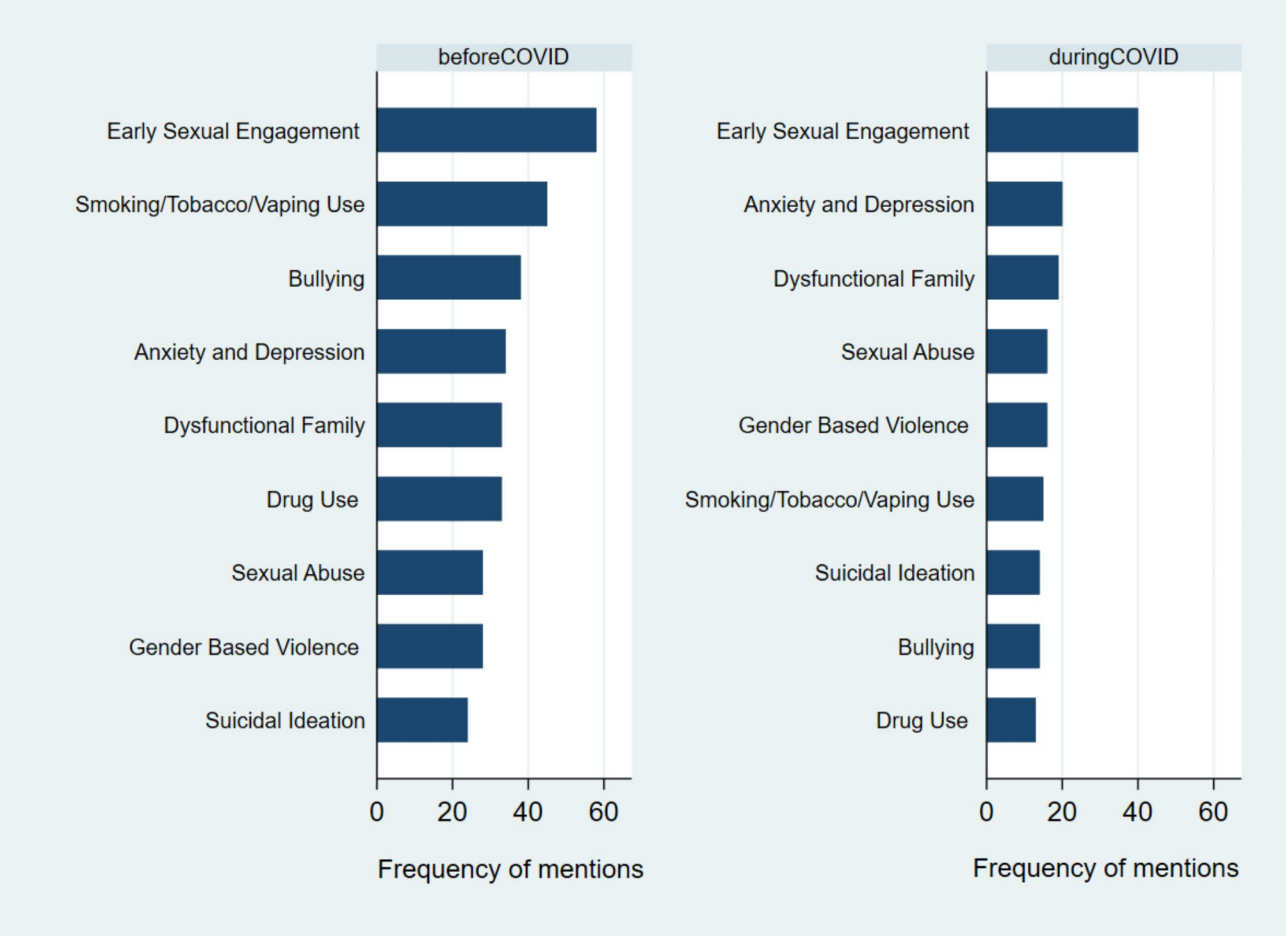
Psychosocial concerns among adolescents before and during the COVID-19 pandemic

Table 1 and Fig. 2 shows the frequency of identified psychosocial concerns among adolescents as reported by health facilities. The ranking within categories of concerns before and during COVID-19 provide a descriptive assessment of changes due to COVID-19 (Fig. 2). Before and during COVID-19, the most mentioned psychosocial among adolescents served by health facilities was early sexual engagement. This therefore ranked first, with 58 facilities and 40 facilities reporting this before and during COVID-19. The order of frequency before COVID-19 was Early Sexual Engagement, Smoking/ Tobacco/ Vaping Use, Bullying, Anxiety and Depression, Drug Use, Dysfunctional Family, Gender Based Violence, Sexual Abuse, Suicidal Ideation. During COVID-19, there was change in the ranking with the order as shown in Table 1. Two months into quarantine, when this survey was conducted, the top concerns in addition to Early Sexual Engagement were now Anxiety and Depression, Dysfunctional Family, Sexual Abuse and Gender Based Violence.

### Phase II: regression results

Table 2 presents the results of ordered logistic regression of AHS utilization (number of adolescents served per week) on staff availability, staffing disruptions and number of service provision modalities. We ran sequential models and report results from the final model (Model 4).

**Table 2:** Ordered Logistic Regression Models for “Adolescent health utilization (Average number of adolescents provided with health services per week)” [N = 148]

VARIABLES		Model 1	Model 2	Model 3	Model 4
Number of adolescent-friendly care trained staff reporting for duty (Ref: Much less than before)	Less than before	1.102			1.117
		(1.357)			(1.194)
	Same as before	10.25**			5.911*
		(11.80)			(5.898)
Experiencing disruptions in your usual staffing that provide adolescent health services	[Yes (1) vs. No (0)]		0.186***		0.302**
			(0.0757)		(0.141)
Number of modalities of adolescent service provision in your health center (Ref: 1 modality)	2.modalities			1.890	2.213*
				(0.819)	(1.027)
	3.modalities			2.793**	2.851**
				(1.385)	(1.438)
	4.modalities			5.214***	4.913***
				(2.356)	(2.572)
*Observations*		*144*	*145*	*145*	*144*

Odds ratios shown

*** p < 0.01, ** p < 0.05, * p < 0.1; robust standard errors in parentheses

Regressions included island region fixed effects

Having the same number of adolescent-friendly care trained staff reporting for duty as before COVID-19 is significantly associated with a six-times increase in the odds of having a higher level of AHS utilization compared to having much less staff than before. Facilities that experienced staffing disruptions are 70% less likely to have a higher level of AHS utilization compared to those who reported no staffing disruptions. Using all four modalities is significantly associated with a 4.9 times increase in the odds of having a higher level of AHS utilization compared to using only one modality. Using three modalities is significantly associated with a 2.9-times increase in the odds of having a higher level of AHS utilization while using two modalities is associated with a 2.2-times increase in the odds of having a higher level of AHS utilization (but only marginally significant).

### Phase III: qualitative findings

The most common challenges cited by health facilities included client quarantine restrictions, limitations on adolescent-friendly care trained staff, limited access to public transportation and decreased number of staff. The most common solutions being considered or already implemented were mobilizing barangay health workers (BHW) and health service providers into communities, innovating ways to disseminate information, seeking support from local government, and providing more telehealth opportunities.

*Challenges* Facilities that discussed challenges with quarantine restrictions often mentioned that adolescents were not permitted to leave their houses under the community quarantines and were not given quarantine passes. This affected the ability of facilities to interact at all with clients in the physical health facility space. Also, respondents reflected on the inadequacy of human resources trained in adolescent-friendly care methods, which has been compounded by the pandemic. Many of the trained staff had been re-assigned to work in COVID-19 wards and were not as available to provide adolescent health services.

Furthermore, public transportation had become limited or unavailable because of the quarantine making physical access to facilities more difficult for both staff and clients. It is therefore not surprising that the respondents mentioned that the number of staff working in clinics at one time has been reduced to ensure social distancing. Respondents also referred to the fact that they were working with a skeletal workforce day to day which complements the descriptive data on staff disruptions that almost 40% of facilities surveyed said they were experiencing.

*Adaptive solutions* A number of facilities mentioned that they had mobilized BHW and health service providers into communities, some of which specifically said they did so to interact with adolescents and provide commodities and information on sexual and reproductive health. In addition, respondents discussed attempts by facilities to use new ways to reach adolescent clients, including SMS and WhatsApp messaging, social media like Facebook and placing posters on sexual and reproductive health topics physically around barangays.

Many facilities also sought support from local government units (LGUs) and barangay leadership for resources such as ambulances and cars to provide transportation. If adolescents needed to come into the health facility, local governments could be notified to support. Several were also advocating with LGUs for more staff and more finances for supplies to prevent stock-outs.

Some facilities also stated that they were providing teleconsultation to clients, which could keep them in touch with adolescents even during the quarantine. In addition, if a BHW or on-duty staff had a question while visiting with a client, adolescent-friendly care trained staff made themselves available over phone to coach them or interact directly with the client. Adolescents could also directly call the facility if they had questions about services or their own health, without an appointment.

## Discussion

These results suggest that two months into the pandemic, over 84% of the surveyed health facilities were operating at the same capacity as at before COVID-19, 79% of adolescent-friendly trained staff were reporting for duty and majority of facilities (64%) reported no staff disruptions. However, only 13% of facilities were on average serving the same number of adolescents or greater than before COVID-19. This is an intuitive finding considering the quarantine restrictions ongoing at the time and consistent with evidence on the impact of COVID-19 restrictions on health access and utilization more generally [18, 34, 35].

In addition, we found that mental health issues had become more prominent concerns among adolescents during the pandemic compared to before COVID-19 with Anxiety and Depression, Dysfunctional Family, Sexual Abuse and Gender Based Violence rising to be among the top five psychosocial concerns after Early Sexual Engagement. Hence top concerns had shifted during the pandemic to include Anxiety and Depression, family dysfunction or dealing with sexual abuse and violence. We also observe that those issues which are associated with peer contact and influence – like bullying, smoking/ tobacco/ vaping and drug use drop in rankings. This could be indicative of the community quarantines in the Philippines with school closures, whereby adolescents were spending more time in the home environment with potential of exacerbating family dysfunction. These findings about top psychosocial concerns among adolescents as shared by their health care providers during the pandemic are consistent with subject matter experts’ predictions of the negative impact of COVID-19 restrictions on adolescent mental health and experiences of violence, especially those under quarantine [29–31, 36]. Literature reviews of studies from China, USA, Brazil and Paraguay have also reported increased rates of anxiety, depression and post-traumatic symptoms among adolescents [37, 38, 39].

Our regression findings demonstrate that more staff reporting for duty is positively correlated with serving more adolescents during the pandemic compared to having much less staff available, thereby suggesting a positive association between staff availability and AHS utilization. On the other hand, facilities that reported staff disruptions were more likely to report serving a smaller number of adolescents compared to those that reported no disruption. This is consistent with our hypothesis as well as evidence on the indirect impact of COVID-19 through shifting of staff to COVID-19 care or limiting the number of staff to allow for social distancing. Our study also elucidates interesting evidence on a potential mitigating mechanism during the pandemic. We show that facilities that use more than one service modality including telemedicine were more likely to report a higher level of ARH service utilization than those who used only one modality (typically having patients/clients come to the facility). This is comparable to findings by a study of a clinic in USA that rapid pivoting to telemedicine allowed for routine visits via video to be conducted with adolescents on a wide variety of topics, including sexual and reproductive health and mental health [40]. While there was a reduction in in-person visits at the beginning of the pandemic, Woods et al. (2020) reported significant increase in telehealth use. A rapid review of national guidelines for COVID-19 in 14 countries including Philippines found recommendations for the use of telehealth or other virtual care modalities for ensuring continuity of essential primary care services [41]. Our study provides evidence that while COVID-19 related staff disruptions are associated with lower utilization of AHS among adolescents; on the other hand, facilities that use more modalities including telehealth and health worker mobilization to engage adolescents are more likely to serve adolescents at the same or even greater level than before the pandemic.

These results are complemented by the qualitative analysis and highlighted some of the common challenges health facilities were facing during the early stages of the pandemic in the Philippines. The most common challenges did align with our quantitative analysis findings and provided some insight into how staffing disruptions and quarantine restrictions were limiting utilization of adolescent health services. They also showed some of the solutions that facilities were deploying as they sought to adapt in the pandemic including telehealth, community health worker mobilization and innovations in information dissemination. The findings from the qualitative analysis seem to imply that mobilized community health workers made use of technology in engaging with adolescents e.g., using telephones to connect adolescents with adolescent-friendly trained staff where necessary. Community health workers (known as Barangay/Village Health Workers in the Philippines) have been considered critical resources for ensuring continuation of essential services and utilization of services during the pandemic as well as in humanitarian settings [42–45]. This study contributes to the growing evidence base about the feasibility of adapting or expanding CHWs’ support to other roles besides primary care provision such as home delivery of medications [46–48]. Our data suggests that barangay health workers were able to connect with adolescents in their household visits during the pandemic, and that facilities also sent health service providers into the community to maintain contact with patients. Further research is needed to understand the cost-effectiveness as well as the impact of this approach on the uptake of healthy behaviors and outcomes among adolescents.

This study has some limitations. One is the self-reported nature of the data which makes it subject to recall bias as respondents were asked to compare current and past events. However, the recall period is of a relatively short duration of three months minimum. The data may also be subject to social desirability bias if respondents subconsciously feel pressure to paint their health facilities in a more positive light. In addition, this is a cross-sectional survey and hence, we can only report correlations and are unable to make causal claims. Also, the facilities in the study may be systematically different from other health facilities which limits the generalizability of the findings to all RHUs or other types of facilities in the Philippines.

Nevertheless, this study through its use of a mixed-methods approach adds to the limited evidence base on how shocks or stressors can impact health utilization among adolescent as well as successful adaptations. We found an increased prominence of mental health needs among adolescents during the pandemic and thus recommend inclusion of increased screening and support for mental health care among adolescents as part of preparedness and response plans during shocks [31, 38, 49, 50]. In addition, the information flow between government child welfare systems and health facilities should be improved to allow for identification and flagging of abuse and violence occurring in households during the pandemic.

Also, even though majority of facilities were maintaining typical operating hours and had most adolescent-friendly care trained staff reporting for duty as before the pandemic as well as up to 64% reporting no staffing disruptions, utilization of AHS was lower than before the pandemic in 87% of surveyed facilities. However, the use of multiple modalities of service provision by facilities is associated with increased utilization of AHS. This implies a need for increased investments in different modalities of service provision, such as telehealth and community health workers training, mobilization and support to help improve AHS utilization outcomes and sustain connection with adolescents during shocks, including a future outbreak or other stressors which could limit physical access to health facilities such as natural disasters.

## Conclusion

This study finds that adolescent health service utilization reduced during the pandemic while adolescent mental health issues became more prominent. The study also adds to the evidence base on potential approaches for adaptations to ensure continued delivery of essential primary care services during shocks such as implementing multiple modalities of service provision including telemedicine and deployment of CHWs [51]. There are several human, organizational, and technical challenges to the implementation and integration of telemedicine in low and middle income country contexts such as in the Philippines including skepticism, financing and reimbursement problems, poor digital training, inadequate infrastructure, and confidentiality and privacy concerns [26, 52]. Hence, stakeholders need to redouble efforts to increase investment in telehealth and other virtual approaches given their immense promise for continuity of health care access during shocks. While emerging infectious disease outbreaks may not be entirely predictable, our findings imply that smart investments in virtual strategies such as telehealth and leveraging of mobile technology can help improve resiliency of health systems in low and middle countries to ensure that previous gains in adolescent health are sustained and existing gaps are addressed.

## Data Availability

The datasets used and analyzed during the current study are not publicly available as they were collected by a donor-funded program that is still ongoing; but are available from the corresponding author on reasonable request.

## List of abbreviations

AH: Adolescent Health
AHS: Adolescent Health Services
ARH: Adolescent Reproductive Health
CHW: Community Health Workers
DOH: Department of Health
AFHF: Adolescent Friendly Health Facilities
